# The impact of COVID-19 perceived threat and restrictive measures on mental health in Italy, Spain, New York, and Hong Kong: An international multisite study

**DOI:** 10.3389/fpsyg.2022.1002936

**Published:** 2022-11-02

**Authors:** Denise Vagnini, Wai Kai Hou, Clint Hougen, Adrián Cano, Andrea Bonanomi, Federica Facchin, Sara Molgora, Francesco Pagnini, Emanuela Saita

**Affiliations:** ^1^Department of Psychology, Università Cattolica del Sacro Cuore, Milan, Italy; ^2^Department of Psychology, The Education University of Hong Kong, Hong Kong, Hong Kong SAR, China; ^3^Centre for Psychosocial Health, The Education University of Hong Kong, Hong Kong, Hong Kong SAR, China; ^4^Gordon F. Derner School of Psychology, Adelphi University, Garden City, NY, United States; ^5^Department of Psychiatry and Clinical Psychology, University of Navarra, Pamplona, Spain; ^6^Department of Statistical Science, Università Cattolica del Sacro Cuore, Milan, Italy; ^7^Department of Psychology, Harvard University, Cambridge, MA, United States

**Keywords:** COVID-19 pandemic, depression, anxiety, physical threat, restrictive measures, economic crisis

## Abstract

In the early stages of the COVID-19 pandemic, Italy, Spain, New York, and Hong Kong stood out for the ir high rates of infections. Given this scenario, a web-based international multisite and cross-sectional study was conducted between April and May 2020 to investigate the psychological impact of the pandemic and the restrictions imposed by the governments in these countries. We expected similar patterns in European countries, and no significant differences in terms of psychological impairment between Hong Kong (with a previous experience related to SARS, but subjected to restrictions for a longer time) and the other areas. Participants were 1955 adults from the above-mentioned areas. We assessed anxiety (GAD-7), depression (PHQ-9), COVID-19-related threats, and perceived burden of restrictive measures. Two-explorative factor analyses (EFAs) with Promax rotation identified COVID-19-related factors: personal physical threat, personal economic threat, global economic threat, and restriction-related burden. ANOVAs studied locations’ differences and two-separate hierarchical multiple regression analyses by location determined whether and how COVID-19-related variables were associated with anxiety and depression, adjusting for age and sex. Italy and Hong Kong showed higher anxiety than Spain (*p* < 0.05); Hong Kong scored higher on depression than Italy and Spain (*p* < 0.001), which highlighted the lowest mean-score. New York participants showed the poorest mental health conditions. Anxiety was predicted by restriction-related burden (β_NY_ = 0.242; β_HK_ = 0.116) and personal economic threat (β_NY_ = 0.246; β_HK_ = 0.145) in New York (Adj.*R*^2^ = 0.125) and Hong Kong (Adj.*R*^2^ = 0.079); by global economic threat (β = 0.199) and restriction-related burden (β = 0.124) in Italy (Adj.*R*^2^ = 0.108); and by personal physical threat (β = 0.144) in Spain (Adj.*R*^2^ = 0.049). Depression was predicted by restriction-related burden (β_NY_ = 0.313; β_HK_ = 0.120) and personal economic threat (β_NY_ = 0.229; β_HK_ = 0.204) in New York (Adj.*R*^2^ = 0.161) and Hong Kong (Adj.*R*^2^ = 0.089); by global economic threat (β = 0.209) in Italy (Adj.*R*^2^ = 0.149); and no predictors emerged in Spain. Findings could contribute to understanding the specific impact of the pandemic on people’s psychological health in each area, along with the factors that impacted mental health. This information may be useful to implementing prevention interventions in case of restrictions.

## Introduction

The COVID-19 outbreak is a global health crisis caused by the novel SARS-CoV-2 virus. It quickly spread between late 2019 and early 2020, leading to many infected people and deaths because of its severity, unknown clinical course, and the possibility of transmission by asymptomatics. It was declared a global pandemic by the WHO on March 11, 2020 (Cucinotta and Vanelli, 2020), and more than 2 years later, it evolves and persists with mutations of the virus and immunization challenges.

Hong Kong was one of the most affected territories from central China, with cases detected as early as January 2020 (Cheuk-Man Li, 2020). By early spring 2020, Italy and Spain were among the epicenter states of the virus in Europe (Celyan, 2020; Mazza et al., 2020; Rodrìguez-Rey et al., 2020; Gualda et al., 2021), and similarly, New York became one of the areas most affected in North America (Thompson et al., 2020).

To contain the spread of COVID-19 infections and prevent the hospitals’ collapse, governments enacted restrictive measures, at first limited to the most affected areas, but eventually extended to entire countries worldwide. Regulations consisted of different quarantine measures to avoid social/physical contact and to reduce exposure to contagion.

These regulations were implemented by central and local authorities in different ways in China, European nations (such as Italy and Spain), and the United States (especially New York). Some areas were more successful in flattening the curve of infections than others, enacting a rapid response to the first batch of cases. By January 23, 2020, the Hong Kong government moved quickly to set up quarantine centers, border restrictions, and other preventive measures throughout the densely populated area. Initially these rules (e.g., social distancing, hand washing, and masking) were less restrictive and preventive, but from the end of March to the end of May 2020, restrictions on non-essential commercial activities, school closures, and stricter remote or no-contact working rules were put in place. Contact tracing and quarantine requirements for clusters of COVID-19-infected people were enforced, but lower-risk outdoor activities continued to be permitted and a full-fledged lockdown was avoided. Subsequently, after confirmation from the WHO of the virus’ transmission outside of China, restrictive strategies, which varied according to differences in health care infrastructure, were gradually applied as a global response (Khanna et al., 2020; Koh et al., 2020; Ren, 2020; Cheung et al., 2021).

European countries initially delayed the implementation of strict preventive measures and failed to anticipate new cases. In Italy, only a cluster of northern cities in “red zones” (i.e., regions of Lombardy and Veneto) were subjected to lockdown protocols. After March 8, 2020, these measures were extended to the whole nation because of the overwhelming volume of critical cases and their impact on the healthcare system. This first phase included prolonged confinement at home. Italian citizens with a self-certification document to show to law enforcement could leave the house only for reasons related to work or health, or to purchase supplies. The President of the Council of Ministers of the Italian Republic issued several decrees that extended these containment measures until May 18, 2020, followed by various reopening ordinances.

Similarly, on March 16, 2020, Spain imposed lockdown measures due to a sudden increase in cases, and, as in Italy, this included a shutdown of non-essential businesses and stay-at-home orders. Subsequent more restrictive extensions stretched these measures until late May (Khanna et al., 2020; Tobías, 2020).

In the United States, President Trump declared a national emergency on March 13, 2020. Different restrictive measures according to local politics and the pandemic’s status across different states were implemented. In New York, residents were asked to stay at home except to accomplish essential tasks (i.e., grocery shopping), while workers in essential businesses continued to attend in-person. State-level lockdown rules (e.g., suspension of mass transport and industrial activities) began on March 22, 2020, with extensions until May 15, 2020. In early April, the United States became the country with the largest number of COVID-19 infections, with New York as the epicenter. Hospitals were saturated and running out of medical devices and supplies needed to cope with the emergency (Khanna et al., 2020; Shehzad et al., 2020).

During this health emergency, restrictive measures were necessary to bring the R-index<1 (number of people who could be infected by an infected person) with the use of non-pharmaceutical interventions to decrease hospitalizations.

### Background

COVID-19 confinement measures had considerable adverse effects on people’s psycho-social health (Clemente-Suárez et al., 2020; Rossi et al., 2020; Hou et al., 2021).

Researchers found that the burdensome nature of mass quarantine, isolation, loneliness, and concerns for the health of loved ones and one’s self could lead to increased levels of fear, stress symptoms, and emotional disturbance, impacting general mental health (Paredes et al., 2021).

Studies conducted worldwide with Asian, European, and American samples revealed that a significant percentage of the population experienced intense emotional distress amidst these life-changing events and associated constant negative stimuli (Clemente-Suárez et al., 2020; Twenge and Joiner, 2020; Wang et al., 2020). A previous study of the Hong Kong general population found that disruptions of daily routines (e.g., engaging in recreational activities, attending work/study) were positively linked to mental health disturbances, with an increase in anxious and depressive symptoms (Hou et al., 2021). Furthermore, a systematic review of studies of eight different countries (including China, Spain, Italy, and the United States) showed that, during the pandemic, relatively high rates of probable anxiety, depression, stress-related disorders, and distress were reported (Xiong et al., 2020). Similarly, recent studies (e.g., Dettmann et al., 2022) found that the first lockdown measures increased the prevalence of psychiatric symptoms (i.e., anxiety and depression) among the general population, compared to pre-pandemic data.

Since the beginning of the pandemic, the magnitude of the tripartite crisis to physical, psycho-social, and economic health become a topic of increasing scientific inquiry. Of particular focus were the long-term consequences on psychological health (Clemente-Suárez et al., 2020). Researchers have focused on the potential factors affecting individuals’ mental well-being in the pandemic context, trying to identify those elements with a key role in mental health symptomatology (Paredes et al., 2021).

Petherick et al. (2021) hypothesized that pandemic fatigue originated both from compliance with preventive measures (i.e., using a mask, sanitizing hands/environments, and limiting the use of public transport) that leave one in a state of hypervigilance and activation, and from exposure to COVID-19-related fake news that circulates on the Internet and stokes confusion, fear, and insecurity. From a physiological perspective, pandemic fatigue is the point at which people move from an acute to a chronic stress experience, and tend to manifest impairment of psychological well-being and adopt unsafe behaviors for their own protection from contagion (Han et al., 2018; Petherick et al., 2021).

This can be explained with the General Adaptation Syndrome (Selye, 1950), which exposes three stress reaction’s phases: alarm, resistance, and exhaustion. Some stressful situations, such as the case of COVID-19-pandemic without precedents in modern history, continue for long periods of time. If the subject struggles to cope with stress in a functional way, the body remains on alert and continues to secrete stress hormones (from the hypothalamic–pituitary–axis). The prolonged exposure to chronic stress can lead to individual, behavioral, and cognitive resources, depletion. The psychophysical effects of this stage weaken the subject’s immune system and are closely related to the development of anxiety, depression, fatigue, and burnout symptoms (Selye, 1950; Lupe et al., 2020).

Furthermore, some studies (e.g., Wise et al., 2020) showed that, while individuals are aware of the personal threat caused by the virus, they could underestimate their personal risk compared to others due to an inclination toward optimism bias. Fear of being infected or dying are common feelings during the outbreak, identified as of the most significant correlates of poor psychological wellness (Bhattacharjee and Acharya, 2020). Furthermore, together with the tension for one’s own well-being, negative psychosocial experiences are associated with the stigma of being, or being able to become, a vehicle for transmission of the virus to others. Stigmatizing people with COVID-19, in fact, could lead to discouraging the adoption of healthy behaviors, underestimating or ignoring symptoms, and experiencing a state of constant distress (Javed et al., 2020; Peprah and Gyasi, 2021). Some researchers (Pérez-Fuentes et al., 2020) defined a “circular relationship” in which the perceived threat influences one’s psychological state leading to a negative mood, and, in turn, this state of activation and fatigue exacerbates thoughts about the threat itself.

The pandemic fomented concerns beyond health concerns. Prolonged closures and decreased business activity stoked fears of uncertainty and economic downturn. The state of emergency stood to impact the economy through two main channels: (1) an increase in demand for medical and essential services, home delivery, remote activities, and information technology and, in contrast, (2) a decrease in demand in fields of tourism, public transportation, catering, entertainment, and so on. In this situation, the imbalance in the economy could foster unprecedented damage with long-term consequences (Sukharev, 2020). The prospect of irreversible economic damage was also a source of psychological distress, particularly in terms of the stress associated with loss of employment, or the fear of losing employment. The fear of no longer being able to support the family or provide for family expenses provided the impetus behind increasing levels of mental illness (Bhattacharjee and Acharya, 2020; Choi et al., 2020).

### Purpose

The aim of this study was to investigate the impact of the (1) COVID-19 pandemic-related perceived threat, and the (2) perception of fatigue with respect to the restrictions imposed by the government on people’s psychological health (i.e., symptoms of anxiety and depression). In addition, we wanted to compare the psychological health (in terms of anxiety and depressive symptoms, COVID-19 pandemic-related perceived threat, and restrictions-related fatigue) among four locations that were initially most affected by the pandemic (i.e., Italy, Spain, New York, and Hong Kong).

Considering the relative closeness in lockdown measures among Italy, Spain, and New York, we expected similar trends in terms of citizen’s psychological well-being (i.e., anxiety and depressive symptoms, COVID-19 pandemic-related perceived threat, and restrictions-related fatigue), especially in Italy and Spain (the two European countries initially most impacted by high COVID-19 infection rates).

As for Hong Kong, according to scientific literature (Cheung et al., 2021), previous experience with the SARS epidemic in 2003 could, in theory, have led the Hong Kong government to find itself better prepared from an organizational point of view, and the population could have initially faced the COVID-19-pandemic through a collective memory of past behavior, which helps to adopt positive health behaviors during catastrophic events or epidemics. However, despite Hong Kong’s prompt response to the first outbreak, this area was under restrictive measures for nearly 2 months longer than the other three locations involved in the study at the time of data collection. Longer quarantine time was associated with the highest symptomatology in terms of psychiatric and stress symptoms (Reynolds et al., 2008; Brooks et al., 2020), so we expected that Hong Kong citizens could be equally exhausted due to the stressful, prolonged restrictive measures, and concerns about the future of the economy, such as the other international sites involved.

Given this scenario, as previous evidence pointed out (e.g., Gan et al., 2020; Muehlschlegel et al., 2021), we expected that this social-and-health emergency could have depleted the psychological resilience resources of Hong Kong’s population, leading to delayed effects on psychological health (e.g., anxiety and depressive symptoms), expressed in the medium-long term of lockdown and quarantine period (i.e., when we collected data in this place).

For these reasons we hypothesized:

*H1*: Similar outcomes in terms of anxiety and depressive symptoms, relations with COVID-19 pandemic-related perceived threat, and restrictions-related fatigue, especially among European countries (i.e., Italy and Spain), and with New York.

*H2*: No psychological impairment’s differences (in terms of anxiety and depressive symptoms, relations with COVID-19 pandemic-related perceived threat, and restrictions-related fatigue) between Hong Kong and the other three locations (i.e., Italy, Spain, and New York).

To sum up, the contributions of this study consist of proposing an overview of the psycho-social well-being of a sample of Italian, Spanish, New York, and Hong Kong citizens during the first spread of COVID-19 infections, and highlighting the role of the COVID-19 perceived threat and restrictions’ related fatigue on the possible development of anxious or depressive symptoms in the four countries involved.

## Materials and methods

### Procedure and participants

This is a cross-sectional, international multisite study conducted between the beginning of April and the beginning of May 2020, during the first COVID-19 outbreak in Italy, Spain, New York, and Hong Kong. In European countries, adult participants from the general population were recruited online with a snowballing sampling method through an invitation to take part in the research posted on social media (i.e., Facebook, LinkedIn, and WhatsApp), or sent to an email contacts list. New York recruitment took place through MTurk, and Hong Kong data were collected *via* Rakuten Insight Surveys using an incentive of 90 Rakuten ePoints (~HKD 9). The total sample was self-selected and non-probabilistic. We could not quantify, as required by the American Association for Public Opinion Research (AAPOR) guidelines, all invitations, republishes through social media, and therefore the response rates of our international multisite survey.

To be included, eligible participants had to be aged ≥18, a resident of one of the four locations involved, with internet access, and able to read and write in languages under study (i.e., Italian, Spanish, English, or Chinese). Data were collected through a self-reported online survey distributed through an anonymous link. On the first page of the survey there was a letter containing the detailed description of the research study, the privacy policy in accordance with the principles of the Helsinki Declaration of 1964 and its later amendments, and a section containing the e-mail contact of the research manager for further information. Accordingly, each participant was apprised about the aims and procedures of the study as expected by the current ethical legislation in force, and had to give informed consent in order to continue with the questionnaire. Preventive multiple submissions options and filter questions were used to avoid duplications and fraud. The survey link could only be opened once by each participant, and the compilation of the initial filter questions was necessary in order to access the survey.

A total of 2,229 people accessed the survey from the four countries, and only those who responded at least partially were included. The total sample was composed of 1,955 participants. Of these, 470 were from Italy, 451 from Spain, 534 from New York, and 500 from Hong Kong.

Using G*Power 3.1 software (Faul et al., 2007, 2009), given α, sample size, and Cohen’s f^2^ effect sizes, the calculation of the achieved power analysis (1-β) is >0.95 (Cohen, 1988).

The study was approved by Ethics Commission of the Department of Psychology at Università Cattolica del Sacro Cuore, Milan, Italy (CERPS: Commissione Etica per la Ricerca in Psicologia), protocol N° 06–20.

### Measures

Self-report online questionnaires were administered to collect socio-demographic data (sex, age, education level, and marital status), clinical (anxiety and depression symptoms), and COVID-19-related variables. All the surveys were either validated versions in respective languages (such as for measures of anxiety and depression), or translated (as was done for *ad hoc* COVID-related items) into the native language of the participants through back-translation technique. This procedure requires a researcher to write the set of items in his source language, and then two bilinguals are employed: one must translate from the source to the target language, and the other blindly translates back from the target to the source. Finally, the researcher must evaluate whether the two final versions in the original language are comparable. If so, it can be reasonably recommended that the target version of the items from the middle of the process expresses the same concept (Brislin, 1970).

The Generalized Anxiety Disorder (GAD-7) (Spitzer et al., 2006) is a 7-item self-report questionnaire rated on a 4-point Likert scale (0 = not at all; 3 = nearly every day) used to measure symptoms of anxiety. According to criteria, a score ≥ 10 indicates clinically significant anxiety symptoms. Cut-off scores are: 0–4 minimal, 5–9 mild, 10–14 moderate, 15–21 severe. Cronbach’s Alpha analysis by country showed a good internal reliability, with 0.89 < α < 0.95.

The Patient Health Questionnaire (PHQ-9) (Kroenke et al., 2001) is a 9-item self-report questionnaire rated on a 4-point Likert scale (0 = not at all; 3 = nearly every day) used to measure the presence of depressive symptoms. A cut-off point of ≥15 defines the presence of significant depressive symptomatology. Other cut-off rates are: 0–4 minimal, 5–9 mild, 10–14 moderate, 15–19 moderately severe, 20–27 severe. Cronbach’s Alpha analysis by country showed a good internal reliability, with 0.83 < α < 0.92.

COVID-19 pandemic-related perceived threat was measured using a battery composed of 9 items on a 4-point Likert scale (1 = strongly disagree; 4 = strongly agree). Example items: “COVID-19 may seriously damage my physical health (e.g., lung function). Cronbach’s Alpha analysis of each factor by country: (F1) personal physical threat 0.77 < α < 0.89; (F2) global economic threat 0.74 < α < 0.83; and (F3) personal economic threat 0.60 < α < 0.73.

The subjective perception of fatigue in complying with the restrictive measures imposed by respective governments was measured through 14 statements. For each item, respondents rated their level of perceived burden on a 5-point Likert scale (1 = not at all; 5 = very much). Example items included “Wear a face mask” and “Avoid crowded places.” Cronbach’s Alpha analysis by country showed a good internal reliability, with 0.88 < α < 0.96.

### Statistical analysis

Data were analyzed using IBM SPSS version 27.0 (IBM Corp. Released, 2020). Not all participants completed the survey. Missing value analysis was conducted to study missing data in terms of frequencies and percentages on each country. Incomplete responses were retained, and no attempt was made to replace missing data.

Descriptive statistics (i.e., frequency, mean, and SD) were performed to present findings for all variables.

Two-separate EFAs were conducted to identify the latent constructs underlying the two COVID-19-related factors questionnaires (concerning pandemic-related perceived threats and perception of fatigue with respect to the restrictions). A Principal Axis factorization extraction method and Promax rotation with Kaiser normalization were used. To determine the goodness of the factorial structure of each extracted dimension, the Kaiser-Mayer-Olkin (KMO) test considering an adequate index >0.5 (Kaiser and Rice, 1974), Bartlett’s test of sphericity (χ2, and *p* value <0.05), and factor loadings (>0.30) were studied. For each extracted factor was reported the explained variance.

Factors’ mean scores were calculated for each respondent by summing ratings for each factor and then dividing by the number of items used to measure it. Asymmetry and kurtosis were studied for each metric variable to demonstrate normal distribution (acceptable values between-1 and + 1). Cronbach’s Alpha coefficient was used to measure internal consistency of each measure with acceptable values ≥0.65 for self-report questionnaires (Vaske et al., 2017).

One-way ANOVAs were performed to determine locations-related differences on anxiety (GAD-7), depression (PHQ-9), and the COVID-19-related factors. Bonferroni correction for *post hoc* pairwise comparison analysis was applied.

A Pearson’s linear correlation coefficient was computed to determine the relationship between COVID-19-related factors and clinical variables (GAD-7; PHQ-9). To identify confounding demographic variables (i.e., sex and age) for subsequent analysis Pearson’s linear correlation and Chi-square tests (α = 0.05, two-tailed) (Cohen, 1988) with anxiety and depression were conducted.

Finally, two-separate hierarchical multiple regression analyses were conducted for each location (i.e., Italy, Spain, New York, and Hong Kong), adding up to a total of 8 regression models. Bonferroni correction with *p* < 0.0125 was applied. In each regression, demographic variables related (*p* < 0.05) to clinical-outcome measures were included in the first step, and a stepwise regression method was used to determine how COVID-19-related factors were associated with anxiety and depression in the second step. Cohen’s f^2^ effect size measure was provided for each model, considering a value ≥0.02 as a small effect size, ≥ 0.15 as medium effect size, and ≥ 0.35 as large effect size according to criteria (Cohen, 1988).

## Results

Missing value analysis showed that in Italy missing data ranged from 0.2% (*n* = 1) to 10.6% (*n* = 50), in Spain from 0.2% (*n* = 1) to 4.4% (*n* = 20), in New York from 0.2% (*n* = 1) to 0.4% (*n* = 2), and in Hong Kong, only one item (i.e., “Limit face-to-face interactions with the co-workers at workplace”) registered 13.5% (*n* = 67) missing responses.

### Descriptive statistics

Participants included 1,955 respondents and the sample size was approximately equally divided between locations. Respondents had a mean age of 38.43 ± 13.96 (range 18–80). The majority was female (60.93%), followed by 38.86% male and 0.21% a gender other than male or female. Regarding marital status, 47.85% of the participants were in a couple, while the rest (52.15%) identified as single. Considering educational level, the majority (70.8%) had a college degree or above.

In the total sample, GAD-7 (skewness = 0.86, SE = 0.06; kurtosis = 0.13, SE = 0.11) and PHQ-9 (skewness = 0.98, SE = 0.06; kurtosis = 0.48, SE = 0.11) had a normal distribution. Since there were missing data, mean scores of clinical variables were computed. Descriptive statistical analysis showed a mean score of 0.93 ± 0.74 on GAD-7, and a mean score of 0.85 ± 0.66 on PHQ-9, which correspond to mild scores on anxiety and depression, respectively. Considering all the participants, 15.50% (*n* = 295) of respondents reported moderate to severe levels of anxiety, and 20.86% (*n* = 397) moderate to severe symptoms of depression. Table 1 summarizes demographics and clinical characteristics by location.

**Table 1:** Socio-demographic and clinical measures by location.

	Italy	Spain	New York	Hong Kong
**Sample size – N**	470	451	534	500
**Mean age ± SD (range)**	42.13 ± 16.45 (18–78)	46.73 ± 12.03 (19–80)	33.0 ± 10.78 (18–73)	33.34 ± 11.08 (18–75)
Sex – N (%)*				
Male	101 (21.54)	179 (39.69)	252 (47.28)	227 (45.40)
Female	368 (78.46)	272 (60.31)	279 (52.35)	271 (54.20)
x-gender	–	–	2 (0.37)	2 (0.40)
**Marital status – N (%)** *				
Single	201 (42.86)	134 (29.71)	295 (55.24)	297 (59.40)
In a couple	236 (50.32)	291 (64.52)	215 (40.26)	193 (38.60)
Divorced	28 (5.97)	20 (4.44)	22 (4.12)	8 (1.60)
Widower	4 (0.85)	6 (1.33)	2 (0.38)	2 (0.40)
**Education Level – N (%)** *				
No formal education	–	–	–	1 (0.20)
Elementary school	2 (0.42)	5 (1.12)	6 (1.12)	1 (0.20)
Secondary school	245 (52.13)	60 (13.42)	28 (5.24)	118 (23.60)
College degree or above	223 (47.45)	282 (85.46)	500 (93.64)	380 (76.00)
**Anxiety: GAD-7 – N (%)** *				
Minimal level	257 (60.47)	328 (72.89)	240 (44.95)	281 (56.43)
Mild level	116 (27.30)	87 (19.33)	157 (29.40)	146 (29.32)
Moderate level	46 (10.82)	28 (6.22)	123 (23.03)	56 (11.24)
Severe level	6 (1.41)	7 (1.56)	14 (2.62)	15 (3.01)
Mean score ± SD**	5.0 ± 3.40	3.84 ± 3.29	5.98 ± 4.40	4.58 ± 3.96
**Depression: PHQ-9 – N (%)** *				
Minimal level	260 (61.32)	312 (69.33)	219 (41.00)	166 (33.33)
Mild level	129 (30.42)	103 (22.90)	114 (21.35)	206 (41.37)
Moderate level	27 (6.37)	29 (6.44)	143 (26.78)	105 (21.09)
Mod. severe level	8 (1.89)	6 (1.33)	53 (9.93)	16 (3.21)
Severe level	–	–	5 (0.94)	5 (1.00)
Mean score ± SD**	4.77 ± 3.23	3.99 ± 3.44	7.34 ± 5.37	6.45 ± 4.40

* Valid percentage.

** Score (0-21 GAD-7; 0-27 PHQ-9) calculated with a proportion from the mean score.

### Explorative factor analyses

Two-separated EFAs were conducted on the total sample (*N* = 1955) using Principal Axis factorization extraction method and Promax rotation with Kaiser normalization. COVID-19 pandemic-related perceived threat can be considered as the result of multiple latent factors. The analysis, based on an eigenvalue criterion of >1.0, yielded three latent factors with 56.83% of explained variance: the first factor with 34.58% of explained variance, the second with 13.91%, and the third with 8.34%. The correlation matrix was good. Bartlett’s test of sphericity was significant (χ^2^ = 6217.697; df = 36; *p* < 0.001), and KMO coefficient was 0.771.

Table 2 shows factor loadings, mean score, SD and reliability of factors (*N* = 1955). Factor 1 contains items 1-2-3-4, Factor 2 contains items 8-9, and Factor 3 contains items 5-6-7. The three factors were named (F1) *personal physical threat*, (F2) *global economic threat*, and (F3) *personal economic threat*.

**Table 2:** Factor loadings of the observed variables in Promax rotation (pattern matrix), descriptive statistics, and reliability of factors (*N* = 1955).

		Factor	
	1	2	3
Item 1. I could die from the disease	0.822		
Item 2. My close social partners may die from the disease	0.623		
Item 3. COVID-19 may seriously damage my physical health (e.g., lung function)	0.853		
Item 4. The treatment for COVID-19 may have side-effects which may seriously damage my long-term physical health	0.715		
Item 5. Treatment costs may become a financial burden for me			0.366
Item 6. This Coronavirus pandemic may create a financial burden for me			0.723
Item 7. I may lose my job under this Coronavirus pandemic			0.703
Item 8. This Coronavirus pandemic may result in negative socio-economic conditions		0.807	
Item 9. This Coronavirus pandemic may create a prolonged and severe effect on the economy of the country		0.785	
Mean (range 1–4)	2.99	3.58	2.82
SD	0.64	0.56	0.69
Cronbach’s alpha	0.834	0.798	0.652

Factors had an approximately normal distribution. The correlation matrix shares moderate, positive, and significant linear inter-factor-correlations: F1-F2 (*r* = 0.232); F1-F3 (*r* = 0.431); and F2-F3 (*r* = 0.299) with a *p* < 0.001.

For the subjective perception of fatigue in complying with the restrictive measures imposed by governments measured with 14 items, a robust mono-factorial structure was produced, based on an eigenvalue criterion of >1.0, with 45.33% of explained variance. The correlation matrix was good. Bartlett’s test of sphericity was significant (χ^2^ = 15565.993; df = 91; *p* < 0.001), and KMO was 0.920. Table 3 shows factor loadings, mean score, SD, and reliability (*N* = 1955). The extracted factor was named *restriction-related burden*, and it had a normal distribution.

**Table 3:** Factor loadings of the observed variables (factor matrix), descriptive statistics, and reliability of the extracted factor (*N* = 1955).

	Factor
	1
Item 1. Wear a face mask	0.559
Item 2. Stay at home if you can	0.606
Item 3. Wash your hands often	0.683
Item 4. Use hand sanitizers	0.662
Item 5. Ventilate home	0.582
Item 6. Avoid public transport	0.590
Item 7. Use bleach or detergent to clean your home	0.663
Item 8. Avoid crowded places	0.781
Item 9. Avoid people who cough, sneeze, or look sick in public	0.695
Item 10. Avoid interactions with service providers (e.g., cashiers, salesperson, or food delivery rider)	0.721
Item 11. Limit face-to-face interactions with co-workers at workplace	0.726
Item 12. Limit face-to-face social gatherings with friends in public	0.738
Item 13. Limit face-to-face social gatherings with relatives in public	0.667
Item 14. Greet without body contact (e.g., handshake, hug)	0.710
Mean (range 1–5)	2.58
SD	0.90
Cronbach’s alpha	0.919

### One-way ANOVAs

Descriptive statistics on clinical (anxiety and depression) and COVID-19-related variables (personal physical threat, personal economic threat, global economic threat, and restriction-related burdens), stratified by location (Italy, Spain, New York, and Hong Kong), and using Bonferroni correction in post-hoc analysis, were obtained. Significant differences emerged.

As hypothesized (Hypothesis 2), means and prevalence of anxiety, depression, and COVID-19-related factors in Hong Kong (HK) did not show a markedly better psychological well-being of this population compared to the other three considered; however, contrary to what is expected (Hypothesis 1), European countries and New York (NY) did not show consistent functioning patterns in all the areas under examination.

Considering COVID-19-related factors, HK (3.28 ± 0.58) scored a higher (*p* < 0.001) personal physical threat than NY (2.94 ± 0.73), Spain (ES) (2.92 ± 0.59), and Italy (IT) (2.83 ± 0.53); no differences were highlighted between IT and ES, but IT scored lower (*p* < 0.05) than NY. Then, HK (3.03 ± 0.64) was perceived higher (*p* < 0.001) in personal economic threat than IT (2.66 ± 0.60) and ES (2.57 ± 0.70); no differences between European locations emerged, but there was a difference with NY (2.98 ± 0.70) (*p* < 0.001). Global economic threat was less perceived (p < 0.05) in HK (3.53 ± 0.54) than IT (3.65 ± 0.53) and (*p* < 0.001) ES (3.83 ± 0.43), but higher perceived (*p* < 0.001) than NY (3.38 ± 0.63); IT scored lower (*p* < 0.001) than ES, and European countries were higher (*p* < 0.001) than NY.

Restriction-related burden more characterized (*p* < 0.001) the experience of IT (2.86 ± 0.80) participants than ES (2.36 ± 0.81) and (*p* < 0.05) NY (2.70 ± 1.16); it was lower (*p* < 0.001) in ES than NY, and in HK (2.39 ± 0.59) than IT and NY. Considering clinical variables, IT (0.95 ± 0.65) and HK (0.87 ± 0.75) showed higher (*p* < 0.05) levels of anxiety than ES (0.73 ± 0.63); although NY (1.14 ± 0.84) showed significantly (*p* < 0.001) higher levels on GAD-7 than the other three locations involved. Regarding the presence of depressive symptoms, HK (0.96 ± 0.65) scored higher (*p* < 0.001) than IT (0.71 ± 0.48) and ES (0.59 ± 0.51); IT recorded more (*p* < 0.05) depressive symptoms than ES; and NY’s population (1.09 ± 0.80) was on average more compromised than the other three: HK (*p* < 0.05), IT, and ES (*p* < 0.001). Table 4 summarizes analyses.

**Table 4:** Descriptive statistics, ANOVAs by location***, and *post hoc* pairwise comparisons.

	*N* (Total)	Mean (SD)				*F* test	Effect size (partial Eta^2^)	Pairwise Comparisons (Bonferroni correction)
		IT	ES	NY	HK			
Personal physical threat	1948	2.83 (0.53)	2.92 (0.59)	2.94(0.73)	3.28 (0.58)	*F*(3, 1944) = 50.376 *p* < 0.001**	0.072	[IT-NY]* [IT-HK; ES-HK; NY-HK]**
Personal economic threat	1939	2.66 (0.60)	2.57 (0.70)	2.98 (0.70)	3.03 (0.64)	*F*(3, 1935) = 57.849 *p* < 0.001**	0.082	[IT-NY; IT-HK; ES-NY; ES-HK]**
Global economic threat	1937	3.65 (0.53)	3.83 (0.43)	3.38 (0.63)	3.53 (0.54)	*F*(3, 1933) = 60.634 *p* < 0.001**	0.086	[IT-HK]* [IT-ES; IT-NY; ES-NY; ES-HK; NY-HK]**
Restriction-related burdens	1936	2.86 (0.80)	2.36 (0.81)	2.70 (1.16)	2.39 (0.59)	*F*(3, 1932) = 37.264 *p* < 0.001**	0.055	[IT-NY]* [IT-ES; IT-HK; ES-NY; NY-HK]**
Anxiety (GAD-7)	1907	0.95 (0.65)	0.73 (0.63)	1.14 (0.84)	0.87 (0.75)	*F*(3, 1903) = 26.725 *p* < 0.001**	0.040	[ES-HK]* [IT-ES; IT-NY; ES-NY; NY-HK]**
Depression (PHQ-9)	1906	0.71 (0.48)	0.59 (0.51)	1.09 (0.80)	0.96 (0.65)	*F*(3, 1902) = 61.884 *p* < 0.001**	0.089	[IT-ES; NY-HK]* [IT-NY; IT-HK; ES-NY; ES-HK]**

*** IT, Italy; ES, Spain; NY, New York; HK, Hong Kong;

** *p* < 0.001;

* *p* < 0.05.

### Correlations

Pearson’s linear correlations showed that clinical variables (anxiety and depression) were significantly associated with most of the threat-factors and restriction-related burdens across the four locations (see Table 5). Considering demographics, age showed a significant (*p* < 0.001) negative linear correlation with anxiety (*r* = −0.198) and depression (*r* = −0.295). Chi-square test proved that sex had a significant (*p* < 0.001) correlation with anxiety (χ^2^ = 179.544; df = 75; Cramer’s V = 0.177; Phi = 0.307) and depression (χ^2^ = 283.590; df = 156; Cramer’s V = 0.223; Phi = 0.386).

**Table 5:** Correlation matrix between anxiety (GAD-7), depression (PHQ-9), and the factors personal physical threat, personal economic threat, global economic threat, and restriction-related burden by location***.

		Anxiety				Depression
		IT	ES	NY	HK	IT	ES	NY	HK
Personal physical threat	Pearson Corr.	0.106*	0.130*	0.147**	0.088*	0.092	0.066	0.073	0.115*
	*p* value	0.029	0.006	<0.001	0.050	0.059	0.166	0.092	0.010
	*N*	423	449	534	498	423	449	534	498
Personal economic threat	Pearson Corr.	0.128*	0.133*	0.252**	0.160**	0.126*	0.121*	0.234**	0.216**
	*p* value	0.008	0.005	<0.001	<0.001	0.010	0.010	<0.001	<0.001
	*N*	421	448	534	498	421	448	534	498
Global economic threat	Pearson Corr.	0.217**	0.002	0.065	0.001	0.214**	0.040	0.020	−0.003
	*p* value	<0.001	0.968	0.133	0.990	<0.001	0.394	0.653	0.941
	*N*	419	446	534	498	419	446	534	498
Restriction-related burden	Pearson Corr.	0.147*	0.087	0.247**	0.122*	0.155*	0.099*	0.319**	0.119*
	*p* value	0.003	0.069	<0.001	0.006	0.002	0.038	<0.001	0.008
	*N*	417	442	534	498	416	442	534	498

*** IT, Italy; ES, Spain; NY, New York; HK, Hong Kong;

** *p* < 0.001 (two-tailed);

* *p* < 0.05 (two-tailed).

### Hierarchical multiple regression analyses

Factors extracted from independent-EFAs (personal physical threat, personal economic threat, global economic threat, and restriction-related burden) were used as independent variables in eight hierarchical multiple regression models to test, for each location, the relationship with clinical measures (anxiety and depression). For each regression, age and sex (recoded in dummy variable) were included in the first step, while in the second step, a stepwise method was used. Bonferroni correction with *p* < 0.0125 was applied to test each model.

For NY and HK, analyses showed similar patterns for the effect of predictors on symptoms of anxiety and depression. However, in IT and ES the models worked differently between countries and with respect to the outcome variables. This result disconfirms our initial hypothesis (Hypothesis 1) that specifically European countries could exhibit similar patterns of functioning; however, it claims similar expected functioning (Hypothesis 2) between Hong Kong and another area (NY).

Regression models indicated that in NY (Adj. *R*^2^ = 0.125) and HK (Adj. *R*^2^ = 0.079) anxiety was positively associated with restriction-related burden (β_NY_ = 0.242; β_HK_ = 0.116) and personal economic threat (β_NY_ = 0.246; β_HK_ = 0.145). Model of IT (Adj. *R*^2^ = 0.108) showed that anxiety was predicted by global economic threat (β = 0.199), and restriction-related burden (β = 0.124). In ES (Adj. *R*^2^ = 0.049) personal physical threat (β = 0.144) impacted on anxiety. Table 6 shows results of the best hierarchical multiple regression models emerged by location, indicating how COVID-19-related factors were associated with GAD-7. Cohen’s f^2^ effect size measures (Cohen, 1988) are described in detail.

**Table 6:** Hierarchical multiple regression models^a^.

			Standardized coefficients (Beta)	***t***	Sign.	Adjusted *R*^2^	Effect size (Cohen’s f^2^) ^c^
ITALY	*F*(1, 397) = 6.672 *p* = 0.010	(Constant)	NA^b^	−1.641	0.102	0.108	0.364
		Age	−0.149	−3.130	0.002		
		Sex	0.162	3.415	0.001		
		Global economic threat	0.199	4.185	<0.001		
		Restriction-related burden	0.124	2.583	0.010		
	Excluded variables						
		Personal physical threat	0.088	1.831	0.068		
		Personal economic threat	0.097	1.985	0.048		
SPAIN	*F*(1, 434) = 9.105 *p* = 0.003	(Constant)	NA^b^	1.923	0.055	0.049	0.243
		Age	−0.145	−2.965	0.003		
		Sex	0.112	2.328	0.020		
		Personal physical threat	0.144	3.017	0.003		
	Excluded variables						
		Global economic threat	−0.053	−1.111	0.267		
		Restriction-related burden	0.083	1.765	0.078		
		Personal economic threat	0.091	1.895	0.059		
NEW YORK	*F*(1, 526) = 35.019 *p* < 0.001	(Constant)	NA^b^	−0.250	0.803	0.125	0.388
		Age	−0.105	−2.566	0.011		
		Sex	0.044	1.077	0.282		
		Restriction-related burden	0.242	5.918	<0.001		
		Personal economic threat	0.246	6.035	<0.001		
	Excluded variables						
		Personal physical threat	0.058	1.201	0.230		
		Global economic threat	−0.063	−1.313	0.190		
HONG KONG	*F*(1, 493) = 7.197 *p* = 0.008	(Constant)	NA^b^	1.649	0.100	0.079	0.307
		Age	−0.209	−4.808	<0.001		
		Sex	0.017	0.382	0.703		
		Restriction-related burden	0.116	2.683	0.008		
		Personal economic threat	0.145	3.332	<0.001		
	Excluded variables						
		Personal physical threat	0.014	0.279	0.781		
		Global economic threat	−0.028	−0.600	0.549		

Dependent variable: anxiety (GAD-7).

a Data are adjusted for age and sex.

b NA, not applicable.

c Cohen’s f^2^ standardized measure of effect size (Cohen, 1988): ≥ 0.02 small effect; ≥ 0.15 medium effect; ≥ 0.35 large effect.

Considering depression as a dependent variable, restriction-related burden (β_NY_ = 0.313; β_HK_ = 0.120) and personal economic threat (β_NY_ = 0.229; β_HK_ = 0.204) were predictors in NY (Adj. *R*^2^ = 0.161) and HK (Adj. *R*^2^ = 0.089) models. Global economic threat (β = 0.209) was the only factor predicting depression symptoms in IT (Adj. *R*^2^ = 0.149); no predictors were identified in ES. Table 7 summarizes how COVID-19-related factors were associated with PHQ-9 by location, providing Cohen’s f^2^ effect size measures (Cohen, 1988).

**Table 7:** Hierarchical multiple regression models^a^.

			Standardized coefficients (Beta)	*t*	Sign.	Adjusted *R*^2^	Effect size (Cohen’s f^2^)^c^
ITALY	*F*(1, 398) = 20.520 *p* < 0.001	(Constant)	NA^b^	−0.366	0.715	0.149	0.429
		Age	−0.266	−5.754	<0.001		
		Sex	0.189	4.093	<0.001		
		Global economic threat	0.209	4.530	<0.001		
	Excluded variables						
		Personal physical threat	0.092	1.961	0.051		
		Restriction-related burden	0.108	2.328	0.020		
		Personal economic threat	0.115	2.443	0.015		
SPAIN		(Constant)	NA^b^	6.049	<0.001		
		Age	−0.239	−5.096	<0.001		
		Sex	0.140	2.987	0.003		
	Excluded variables						
		Personal physical threat	0.101	2.188	0.029		
		Global economic threat	−0.008	−0.166	0.868		
		Restriction-related burden	0.110	2.409	0.016		
		Personal economic threat	0.110	2.400	0.017		
NEW YORK	*F*(1, 526) = 32.902 *p* < 0.001	(Constant)	NA^b^	−0.098	0.922	0.161	0.448
		Age	−0.132	−3.294	0.001		
		Sex	0.030	0.750	0.454		
		Restriction-related burden	0.313	7.815	<0.001		
		Personal economic threat	0.229	5.736	<0.001		
	Excluded variables						
		Personal physical threat	−0.023	−0.493	0.623		
		Global economic threat	−0.102	−2.196	0.029		
HONG KONG	*F*(1, 493) = 7.719 *p* = 0.006	(Constant)	NA^b^	1.341	0.181	0.089	0.326
		Age	−0.178	−4.116	<0.001		
		Sex	0.027	0.633	0.527		
		Restriction-related burden	0.120	2.778	0.006		
		Personal economic threat	0.204	4.709	<0.001		
	Excluded variables						
		Personal physical threat	0.014	0.282	0.778		
		Global economic threat	−0.058	−1.262	0.208		

Dependent variable: depression (PHQ-9).

a Data are adjusted for age and sex.

b NA, not applicable.

c Cohen’s f^2^ standardized measure of effect size: ≥ 0.02 small effect; ≥ 0.15 medium effect; ≥ 0.35 large effect.

## Discussion

The objective of this study was to investigate the impact of COVID-19 pandemic-related perceived threats and the perception of fatigue with respect to the restrictions imposed by the government on people’s mental health (i.e., symptoms of anxiety and depression). In addition, we wanted to compare the psycho-social states among four locations that were initially most affected by the pandemic (i.e., Italy, Spain, New York, and Hong Kong). Participants were equally distributed and missing data negligible. Only one item in Hong Kong (i.e., fatigue in limiting face-to-face interactions with co-workers), recorded 13.5% of missing responses. Since it never achieved a full-lockdown, those who missed the item may have had jobs that were not remotely-adapted and they may not have identified with the item.

Our findings could aid in understanding international multisite differences and what has most impacted the psychological well-being of the locations involved. Furthermore, we hope that our findings might provide individuals and mental health professionals with ideas on how to mitigate the psychological effect of the ongoing COVID-19-pandemic and associated restrictions, as well as aid in being better prepared for possible future similar situations.

Literature on the pandemic and people’s perceptions of threat has largely emphasized concerns around physical health and personal and global economic concerns (Bhattacharjee and Acharya, 2020; Choi et al., 2020; Pérez-Fuentes et al., 2020; Sukharev, 2020). In our study, primary threat-factors extracted from the EFA echo this sentiment concerning the perception of threat across all four locations, including concerns about physical threat, personal economic threat, and global economic threat. Given that our data was collected shortly after the first wave of the pandemic, high mean scores suggest that these perceptions of threat were immediate concerns among the involved populations.

In our study, we measured the perceived burdensome nature of complying with preventative measures as a proxy of the construct of pandemic fatigue, widely described in scientific literature (e.g., Petherick et al., 2021; Filindassi et al., 2022). Given that our data was collected after the first wave of the pandemic, we did not yet anticipate the extent to which fake news might guide individual’s decision-making or impact their psychological well-being. An EFA of the perceived burdensome nature of adopting preventative measures produced a unifactorial construct of restriction-related burden. This suggests that in the four locations where we gathered data, the burdensome nature of preventative measures was seen as a whole and already evident after the first wave of the pandemic, since restrictive measures brought drastic and sudden changes in daily life (e.g., Mækelæ et al., 2020; Filindassi et al., 2022).

Perceptions of threat and the burdensome nature of preventative measures were associated with higher levels of depressive symptoms, but their associations varied by location. Not surprisingly (reflecting much of the literature on the pandemic), perceived personal physical threat was associated with anxiety in every location, but only with depression in Hong Kong. Personal economic threat was associated with both anxiety and depressive symptoms in every location, suggesting that it was the pandemic’s economic consequences that most undermined individual well-being. This difference, between personal physical threat and personal economic threat, may be a reflection of the above-mentioned optimism bias as noted by Wise et al. (2020), who also collected their data early in the pandemic.

Perceptions of threat to the global economy were less associated with poorer mental health outcomes, except in Italy. Our study’s proxy for pandemic fatigue, perceived restriction-related burden, was associated with depressive symptoms in all locations and with anxiety symptoms everywhere but Spain.

Significant regional differences existed between every location and every independent variable. There appears to be few trends among the four locations with the exception of New York and Hong Kong. This may point to a limitation in the study given that respondents from New York and Hong Kong were surveyed in major metropolitan areas, whereas in Italy and Spain, respondents came from across the countries in both urban and rural locations. In Italy, perceptions of the global economic threat were mostly likely to predict both anxiety and depressive symptoms. Restriction-related burden also predicted anxiety among Italian respondents. Contrarily, in Spain, only personal physical threat predicted anxiety symptoms, and no perceived threat significantly predicted depressive symptoms. In terms of predictors of mental health outcomes, New York and Hong Kong followed the same trend where in both locations, restriction-related burden and personal economic threat most significantly predicted both symptoms of anxiety and depression. Differences in outcomes among the four locations may be numerous. The aforementioned differences in sampling may account for this in part, but do not explain the differences between Italy and Spain. In Italy, respondents appeared most impacted by the global scale of the pandemic. Spain was the only location where the perceived threat to physical health was a predictor of poorer mental health outcomes. The most apparent similarities were between New York and Hong Kong. This could be a reflection of the more similar sampling approaches in these two locations (both being large, metropolitan areas). Given the respondents emphasis on restriction-burden and personal economic threat, similarities might also be a reflection of the relative importance of commerce and individualism in these two locations. However, while outside the scope of this study, the political differences could also help explain the findings and justify further research in this area, in order to understand their relationship with the development of psychiatric symptoms (e.g., anxiety and depression) among citizens from different sites in contexts of health emergency.

### Limitations

As one of the goals of this study was to examine perceived threats after the first wave of the pandemic, we were limited by the different timing of the COVID-19 virus’ reach to each location. Respondents in Hong Kong were surveyed further after the initial outbreak compared to other locations. This may not have impacted our data significantly as data from Hong Kong paralleled that in New York despite the differences in sampling timing.

Then, we know that participants do not represent a statistically representative sample, and we show our awareness on the differential recruitment methods used. As previous evidence showed (e.g., James and Bolstein, 1990; Singer and Ye, 2012), when respondents received financial compensation there could be a response bias. In our study, benefits of incentives may have increased the likelihood of participation and the degree of effort expended in completing the survey in some areas than others. Finally, this research carries with it the inherent limitations of a cross-sectional study that recruited a convenience sample. Our data points to associations and predictors between variables, and we cannot say with certainty the extent to which the data represents the populations of each location. The overrepresentation of female respondents (particularly in Italy and Spain) presents a limitation to the representative nature of the sample.

## Conclusion

Our study demonstrates the initial impact of the COVID-19 pandemic in four regions. Unlike our prediction, we did not find significant similarities between Italy and Spain, perhaps underscoring the considerable cultural and political differences between the two European countries. Italians took a more global perspective of the pandemic as the threat to the global economy most predicted poor mental health outcomes. In contrast to the other locations, only in Spain did the perceived personal physical threat of the COVID-19 virus predict poor mental health. According to our original hypothesis, Hong Kong did not show better well-being. Despite what was expected, New York was more similar to Hong Kong than to European countries, showing similar patterns where poor mental health outcomes in both metropolises were predicted by restriction-related burden and personal economic threat.

### Practical and theoretical implications

These findings could provide useful information on how to understand the state of psycho-social health of the involved locations (Italy, Spain, New York, and Hong Kong) during the first spread of COVID-19 infections along with the factors which played a crucial role on well-being of citizens, despite the lack of a statistical representative sample.

Awareness of the prevalence of anxiety and depression, and factors (i.e., physical and economic perceived threat, or restriction-related fatigue) that could predict this symptomatology, could be useful for policy development, both in case of other restrictions and health virus-related threats in the future, and to manage actual mental health crisis issues as an inevitable consequence of the pandemic period. Our study could contribute to providing a theoretical foundation for policymakers and mental health services to monitor, guide preliminary support, and direct the implementation of community care and nation-specific or international interventions aimed at safeguarding the population’s well-being.

## Data availability statement

The raw data supporting the conclusions of this article will be made available by the authors, without undue reservation.

## Ethics statement

The studies involving human participants were reviewed and approved by the Ethics Commission of the Department of Psychology at Università Cattolica del Sacro Cuore, Milan, Italy (CERPS: Commissione Etica per la Ricerca in Psicologia), protocol N° 06–20. The patients/participants provided their written informed consent to participate in this study.

## Author contributions

DV, WKH, CH, and ES were involved in literature research, data analysis, drafting, and reviewing the manuscript. AB was involved in data analysis and data interpretation. AC, FF, SM, and FP were involved in drafting and reviewing the manuscript. All authors contributed to the article and approved the submitted version.

## Conflict of interest

The authors declare that the research was conducted in the absence of any commercial or financial relationships that could be construed as a potential conflict of interest.

## Publisher’s note

All claims expressed in this article are solely those of the authors and do not necessarily represent those of their affiliated organizations, or those of the publisher, the editors and the reviewers. Any product that may be evaluated in this article, or claim that may be made by its manufacturer, is not guaranteed or endorsed by the publisher.
